# Specificity of archaeal caspase activity in the extreme halophile *Haloferax volcanii*

**DOI:** 10.1111/1758-2229.12010

**Published:** 2012-11-30

**Authors:** Mansha Seth-Pasricha, Kelly A Bidle, Kay D Bidle

**Affiliations:** 1 Institute for Marine and Coastal Sciences, Rutgers UniversityNew Brunswick, NJ, USA; 2 Department of Biology, Rider UniversityLawrenceville, NJ, USA

## Abstract

Caspase-like proteases are key initiators and executioners of programmed cell death (PCD), which is initiated by environmental stimuli and manifests in organisms ranging from unicellular microbes to higher eukaryotes. *Archaea* had been absent from the caspase inheritance discussion due to a lack of gene homologues. We recently demonstrated extremely high, basal caspase-like catalytic activity in the model haloarcheon, *Haloferax volcanii*, which was linked to the cellular stress response and was widespread among diverse *Archaea*. Here, we rigorously tested the catalytic specificity of the observed archaeal caspase-like activities using hydrolytic assays with a diverse suite of protease substrates and inhibitors compared with known model serine and cysteine proteases (trypsin, cathepsin, papain, and human caspase-8). Our experiments demonstrate that exponentially growing *H. volcanii* possesses a highly specific caspase-like activity that most closely resembles caspase-4, is preferentially inhibited by the pan-caspase inhibitor, zVAD-FMK, and has no cross-reactivity with other known protease families. Our findings firmly root the extremely high levels of caspase-like activity as the dominant proteolytic activity in this extreme haloarcheaon, thereby providing further support for housekeeping functions in *Haloarchaea*. Given the deep archaeal roots of eukaryotes, we suggest that this activity served as a foundation for stress pathways in higher organisms.

## Introduction

Caspases are a family of highly refined, intracellular cysteine proteases that cleave a wide variety of substrate proteins at the C-terminus of an aspartate residue within specific tetrapeptide motifs. They generally display strong conservation in amino acid sequence, structure and substrate specificity (Cohen, 1997; Stennicke and Salvesen, 1998; Thornberry and Lazebnik, 1998) possessing a conserved domain structure with a histidine- and cysteine-containing catalytic diad. A variety of caspases have been identified in different metazoan animals, ranging from *Hydra* to humans (Thornberry and Lazebnik, 1998; Cikala *et al*., 1999; Sanmartín *et al*., 2005) that are key initiators and executioners of programmed cell death (PCD) or apoptosis, a genetically controlled, irreversible form of cell death that elicits specific morphological changes initiated by environmental stimuli (Lockshin and Williams, 1965; Kerr *et al*., 1972).

Although PCD was first discovered in multicellular organisms, which use it for development and defence, it has now been shown to be a ubiquitous trait throughout nature, spanning diverse prokaryotes, and both unicellular and multicellular eukaryotes. PCD is catalysed either by classic caspases or orthologous proteins, such as metacaspases, paracaspases, phytaspases, and saspases (Vaux and Korsmeyer, 1999; Koonin and Aravind, 2002; Coffeen and Wolpert, 2004; Riedl and Shi, 2004; Salvesen and Abrams, 2004; Sanmartín *et al*., 2005; Chichkova *et al*., 2010; Mace *et al*., 2010; Vartapetian *et al*., 2011). Genomic, morphological and biochemical evidence of caspase-mediated PCD has also been documented in widely diverse evolutionary lineages of prokaryotic and eukaryotic phytoplankton including cyanobacteria, coccolithophores, diatoms, and dinoflagellates (Vardi *et al*., 1999; Segovia *et al*., 2003; Berman-Frank *et al*., 2004; Bidle and Falkowski, 2004; Moharikar *et al*., 2006; Bidle *et al*., 2007; Bidle and Bender, 2008). These cumulative findings have peaked interest in exploring the molecular evolution of caspase-like proteins, their function, and the evolutionary drivers that have influenced their retention in different microbial lineages (Ameisen, 2002; Koonin and Aravind, 2002; Bidle and Falkowski, 2004).

Until recently, *Archaea* were absent from the discussion on the establishment, maintenance, and inheritance of apoptotic machinery, as their genomes lack clear homologues of these proteins (Koonin and Aravind, 2002). However, the incidence and roles of caspase-like proteins are only now beginning to be recognized in this third domain of life. We recently demonstrated that the model haloarcheon, *Haloferax volcanii*, exhibits very high, caspase-8-like (IETDase) activity and expression of immunoreactive proteins to human caspase-8 antisera, both of which were induced by salt stress and death and were abolished by *in vivo* addition of a broad-spectrum caspase inhibitor (Bidle *et al*., 2010). Caspase inhibition severely impaired cell growth under low and high salt stress, suggesting a critical role in the cellular stress response (Bidle *et al*., 2010). Furthermore, detection of similarly high catalytic activity and expression of immunoreactive proteins in other haloarchaea (*Halorubrum* and *Haloarcula*) and in diverse members of *Euryarchaeota* (the methanogen *Methanosarcina acetivorans* and the hyperthermophile *Pyrococcus furiosus*) and *Crenarchaeota* (the acidophile *Sulfolobus solfataricus*) argue for broad representation within the archaeal domain (Bidle *et al*., 2010).

It is now evident that caspase-like proteins are likely to be present in all domains of life and are a widespread characteristic of unicellular organisms. By playing a role in normal cell function, caspase-like proteases in *Archaea* appear to participate in normal metabolic pathways, broadening their biological roles beyond apoptosis and cell death. Given the deep archaeal roots of eukaryotes (Yutin *et al*., 2008), it is important to further explore the nature of this caspase-like activity in more detail in order to characterize its function and molecular evolution. It may represent a unique evolutionary lineage of these intriguing enzymes. Here, we present a detailed and focused characterization of substrate and inhibitor specificity for the observed caspase-like activities in *H. volcanii* so we can better understand the nature of these catalytic activities. We did not investigate the nature of the immunoreactive proteins to human caspase antibodies (Bidle *et al*., 2010) in this study, since it is based on epitope similarities of denatured proteins and is not reflective of *in vivo* catalytic activities. Further, recent evidence demonstrates that even metacaspases, well-documented caspase orthologues (Uren *et al*., 2000), have altered substrate specificities and are not responsible for observed caspase activities (Vercammen *et al*., 2007). Hence, the respective catalytic and immunoreactive proteins in *H. volcanii* may not be justifiably linked and should be treated as distinct proteins until verified otherwise. Rather, we used a milleu of proteolytic substrates and inhibitors that target distinct classes of proteases, including specific caspases, to specifically determine if the observed caspase-like activity in *H. volcanii* was indeed caspase-specific or was a reflection of a more general manifestation of serine, cysteine, or metallo proteolytic activity. Our findings firmly root extremely high levels of specific caspase activity as the dominant proteolytic activity in this extreme halophilic archeaon.

## Results and discussion

### Specificity of caspase activity in *H. volcanii* cell extracts

**Fig. 1:**
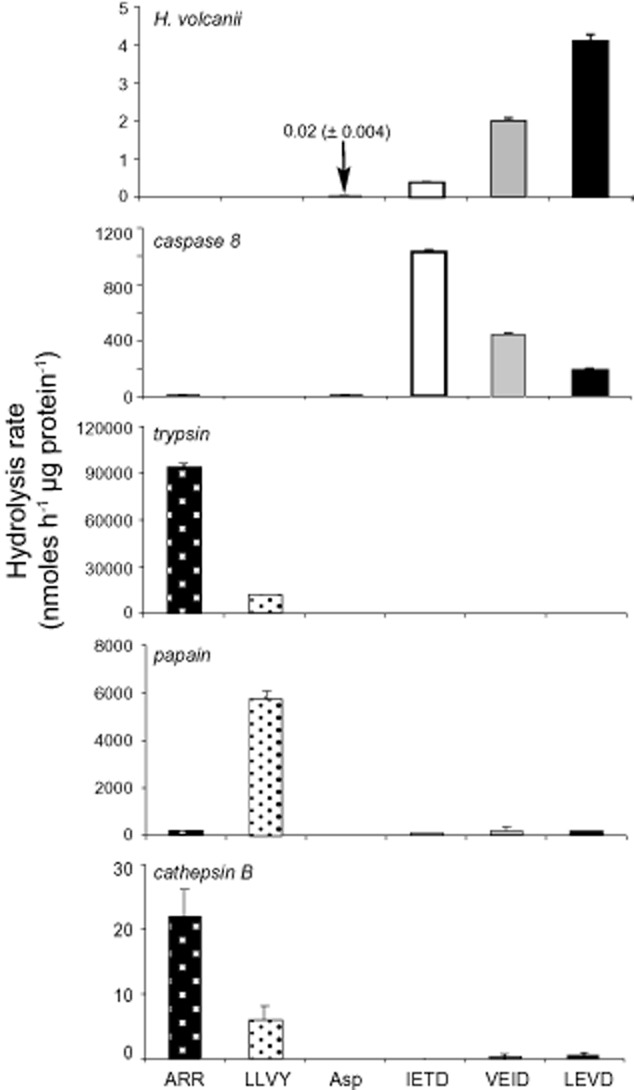
Substrate specificity of *H. volcanii* cell extracts and various purified proteases. *Haloferax volcanii* strain DS70 (Wendoloski *et al*., 2001) was grown aerobically at 45°C in an optimal 2.1 M salt medium (Bidle *et al*., 2008). Cells were harvested from mid-exponential growth and cell extracts were prepared as previously described (Bidle *et al*., 2010) with total protein concentration being determined using the BCA Assay (Thermo Scientific, Rockford, IL, USA). Substrate specificity assays were performed for *H. volcanii* cell extracts, recombinant human caspase-8 (22 ng; BioMol), trypsin (1 ng; Sigma), papain (10 ng; Fisher Scientific), and cathepsin B (250 ng; Sigma), with purified proteases verifying preferred substrate preferences. Cell extracts or purified proteases were incubated with a variety of fluorogenic substrates (final concentration of 100 μM; Sigma): ARR-AMC, LLVY-AMC, Leu-AMC, Asp-AMC *or* canonical tetrapeptide caspase substrates (50 μM final concentration): IETD-AMC (caspase-8), VEID-AMC (caspase-6), LEVD-AMC (caspasae-4; all from Enzo Life Sciences). Assays were performed in 1× Lauber Buffer (50 mM Hepes pH 7.4, 100 mM *NaCl*, 10% Sucrose, 0.1% CHAPS, 10 mM DTT). A modified Lauber buffer consisting of 1.5 M *NaCl* was used for *H. volcanii* cell extract assays (Bidle *et al*., 2010). Kinetic analysis of substrate cleavage was performed (E_x_ 380 nm, E_m_ 460 nm) for 1 h at either 42°C (*H. volcanii* cell extracts only) or 37°C (all other proteases), with readings taken every 3 min using either a Spectra Max Gemini XS or a Spectra Max M3Plate Reader (both from Molecular Devices), using the SoftMax Pro 6.2.1 analysis program. AMC standard calibration curves were performed between 0 and 50 μM and were used to convert RFUs to nmol fluorogenic substrate cleaved. Cleavage rates for *H. volcanii* cell extracts and purified proteases (indicated in each panel) are reported as protein-normalized substrate hydrolysis rates (nmol h^−1^ μg protein^−1^). Individual substrates tested are indicated on the *x*-axis and represented by different bar graph patterns. Error bars represent the standard deviation for triplicate measurements.

**Fig. 2:**
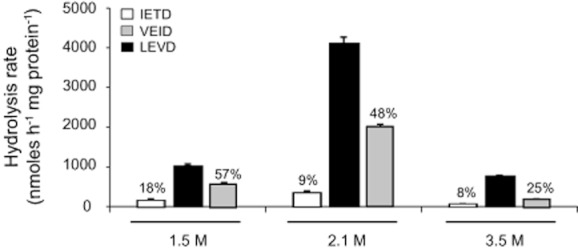
Caspase-specific activities in cell extracts from *H. volcanii* cells grown in low (1.5 M), optimal (2.1 M), and high (3.5 M) NaCl concentrations and harvested at mid-exponential phase. Cell growth at respective salinities was performed as previously described (Bidle *et al*., 2008). Extract preparation and kinetic cleavage assays were conducted as described in Fig. 1, with cell extracts incubating with LEVD-, VEID-, and IETD-AMC. Numbers above VEID and IETD bar graphs indicate the percent of LEVD activity. Error bars represent standard deviations for triplicate measurements.

We challenged cell extracts from exponentially growing *H. volcanii* cells with a diverse suite of canonical, fluorogenic caspase tetrapeptide substrates [YVAD- (caspase-1), VDVAD- (caspase-2), DEVD- (caspase-3), LEVD- (caspase-4), WEHD- (caspase-5), VEID- (caspase-6), IETD- (caspase-8), and LEHD-AMC (caspase-9)] to expand our coverage from previously documented caspase-8-like activity (Bidle *et al*., 2010) and to better elucidate the nature of caspase-like activity in this model haloarchaeon. *Haloferax volcanii* cell extracts displayed ∼ 10- and ∼ fivefold higher specific activities with LEVD-AMC [74 232 relative fluorescence units (RFU) h^−1^ mg protein^−1^] and VEID-AMC (36 001 RFU h^−1^mg protein^−1^) substrates, respectively, compared with IETD-AMC (6909 RFU h^−1^mg protein^−1^). AMC fluorescence calibration curves placed the corresponding substrate hydrolysis rates at 4124, 2000, and 383 nmol h^−1^mg protein^−1^ (Fig. 1). Much lower specific activities were observed for extracts incubating with the other caspase substrates; VDVAD-, YVAD-, DEVD-, LEHD- and WEHD-based activities ranged from only 9% to 47% the IETD-based activity (data not shown). Notably, the same pattern of relative hydrolysis rates for LEVD-, VEID-, and IETD-AMC was observed in extracts from cells grown under low (1.5 M) and high (3.5 M) salt stress; VEID and IETD were 25–57% and 8–18% of these observed LEVD hydrolysis rates (Fig. 2). These findings may indicate the presence of either one or several proteins with overlapping activities and/or functions.

Our observed caspase-like-specific activities are by far the highest ever reported in a unicellular microbe, more notably in an exponentially growing archaeon. Reported caspase-specific (IETDase) activities in diverse unicellular marine phytoplankton, including cyanobacteria, diatoms, coccolithophores, and chlorophytes, are comparatively low for exponentially growing cells [10 s to 100 s of RFUs h^−1^mg protein^−1^ (Segovia *et al*., 2003; Berman-Frank *et al*., 2004; Bidle and Falkowski, 2004; Bidle *et al*., 2007; Bidle and Bender, 2008; Bidle and Kwityn, 2012)], with rates increasing ∼ 10-fold when cells have activated autocatalytic cell death pathways. A wider survey of hydrolysis rates in unicellular microbes using other canonical caspase substrates has only been examined with the unicellular chlorophyte, *Dunaliella tertiolecta* (Segovia *et al*., 2003), whereby hydrolytic cleavage of various caspase substrates was tested in cell extracts from stressed cells that were in different stages of light deprivation (0–5 days). Nonetheless, the maximum activity was seen for IETD and LEHD at ∼ 300 RFU h^−1^mg protein^−1^ after 5 days light deprivation and associated PCD activation. Overall, the activity rates for these substrates ranged from 50 to 300 RFU h^−1^mg protein^−1^ over the 5 day time-course. Our hydrolysis rates for exponentially growing *H. volcanii* cells, along with our previous measurements in diverse *Archaea* (Bidle *et al*., 2010), comparatively dwarf these rates from other unicellular microbes (by more than an order of magnitude), putting them in a unique catalytic and physiological context.

A comparison of substrate specificity for *H. volcanii* cell extracts to four model proteases, trypsin, cathepsin, papain, and recombinant human caspase-8, helped to further place it in the caspase catalytic class (Fig. 1). These model proteases represent distinct classes of peptidases that have well characterized active sites, substrate requirements, and inhibitors (Otto and Schirmeister, 1997; Hedstrom, 2002; Barrett and Rawlings, 2007; Vartapetian *et al*., 2011). Not surprisingly, when challenged with a suite of fluorogenic substrates [ARR-AMC (trypsin and cathepsin), LLVY-AMC (papain), and IETD-AMC, VEID-AMC, and LEVD-AMC (caspase-8, caspase-6, and caspase-4 respectively)], each model protease had the highest activity with its preferred substrate (Fig. 1). Caspase-8 demonstrated notable selectivity among the caspase substrates, with highest catalytic activity for IETD-AMC. We did notice higher IETDase activity in the present study for purified human caspase-8 compared with what had been previously reported (Bidle *et al*., 2010), likely due to a different source and batch of enzyme. *Haloferax volcanii* cell lysates had no measureable activity towards substrates of trypsin, cathepsin, and papain substrates under our incubation conditions at 42°C (Fig. 1).

We also tested for general leucine and aspartate aminopeptidase activity in *H. volcanii* extracts through hydrolysis of leucine-AMC (Leu-AMC) and aspartate-AMC (Asp-AMC), respectively, given the *H. volcanii* genome (http://archaea.ucsc.edu/cgi-bin/hgGateway?db=haloVolc1) has 13 annotated aminopeptidases (HVO_0242, *pepB* aminopeptidase II; HVO_0477, *ampS* aminopeptidase; HVO_0826, deblocking aminopeptidase; HVO_0836, aminopeptidase; HVO_1774, leucyl aminopeptidase; HVO_1829, aminopeptidase homologue; HVO_1849, aminopeptidase; HVO_2600, methionine aminopeptidase; HVO_A0535, aminopeptidase putative; HVO_1966, CAAX aminoterminal protease family; HVO_1997, CAAX aminoterminal protease; HVO_0082 CAAX amino terminal protease; HVO_0160, CAAX amino terminal protease). Leu-AMC is a commonly used aminopeptidase substrate for diverse environmental microbes (Hoppe *et al*., 2002) and has served as a model for general proteolytic activity*. Haloferax volcanii* grows at an acidic pH of ∼ 5.1 and contains ∼ 13 moles of Asp per 100 moles of total amino acids (Mullakhanbhai and Larsen, 1975; Hartman *et al*., 2010), so Asp-AMC incubations helped verify that our observed caspase cleavage were specific to the C-terminal aspartate residue in tetrapeptide motifs, as opposed to a general aspartate cleavage response. Very low Asp-AMC hydrolysis activity (2 ± 0.4 nmol h^−1^mg protein^−1^; Fig. 1) was detected in *H. volcanii* cell extracts, putting it at 0.05% LEVD-ase activity, while no detectable Leu-AMC hydrolysis was detected. For comparison, little to no activity was observed in *Escherichia coli* extracts incubated with Asp-AMC, but Leu-AMC displayed very high activity (2201 nmol h^−1^mg protein^−1^).

Our results confirmed the consistent and extremely high basal activity in exponentially growing *H. volcanii* cells and refined the activity as more caspase-4-like, and demonstrate that the previous findings using IETD actually underestimated *H. volcanii* caspase activity. In order to address the possibility that the absence of some enzyme activities (e.g. LLVY-, ARR-, and Asp-AMC hydrolysis) in our extracts may have been due to competing small molecules, we also independently assayed for these activities in cell extracts heated to 60°C and in partially purified proteins at 42°C obtained using standard ammonium sulfate (AmSO_4_) precipitation techniques (20%, 40%, 60%, 80% and 100%; Fig. S1; Englard and Seifer, 1990). *Haloferax volcanii* has two annotated serine proteases (Hv 1470: Putative intramembrane serine protease; Hv 2225: serine protease) and successful LLVY-AMC cleavage has only been reported at 60°C (Wilson *et al*., 1999), a temperature that is considerably outside the organism's physiological, optimal growth temperature of 45°C (Robinson *et al*., 2005) and more appropriate with temperature stress. Incubation of cell extracts at 60°C did yield LLVYase activity (228 ± 54 nmol h^−1^mg protein^−1^); likewise, LLVYase activity was also observed in the 80% and 100% AmSO_4_ precipitate fractions, albeit much lower (5.7 ± 0.5 nmol h^−1^mg protein^−1^; Fig. S1). Similarly low levels of Asp-AMC hydrolysis to those observed in cell extracts incubated at 42°C (Fig. 1) were measured both in 60°C heated extracts (16.6 ± 0.5 nmol h^−1^mg protein^−1^) and in 80% and 100% AmSO_4_ precipitate fractions (11.6 ± 0.2 nmol h^−1^mg protein^−1^; Fig. S1). While heat treatment did not yield detectable ARR-AMC hydrolysis, relatively high activity was observed in the post-100% AmSO_4_ soluble fractions (Fig. S1). Neither heat-treatment nor AmSO_4_ precipitation recovered Leu-AMC hydrolytic activity. As with cell extracts, caspase activities were by far the highest activities recovered in partially purified protein fractions (Fig. S1); they were generally enriched in 80% and 100% AmSO_4_ fractions and showed the same relative pattern of substrate hydrolysis (LEVDase > VEIDase > IETDase). Boiling of cell extracts at 100°C for 30 min completely abolished all hydrolysis activities, indicative of protein dependence. Our results confirm prior observations (Wilson *et al*., 1999) and provide support for inhibitory molecules of LLVYase activity in cell extracts. Nonetheless, it was particularly striking that LLVYase activity in *H. volcanii* lysates represented only a very small fraction (< 5%) of the maximum observed caspase activity (i.e. LEVDase) and was not detectable at physiological temperatures.

### Selectivity of caspase inhibition

The specificity of caspase-like activity exhibited in *H. volcanii* was further verified by challenging cell extracts with a panel of protease inhibitors that target a diverse array of proteases including serine proteases (phenylmethylsulfonylfluoride, PMSF; aprotinin, Apr), cysteine proteases (leupeptin, Leu; E-64), metalloproteases (EDTA), and a cocktail of these inhibitors (pooled inhibitors, PI) or caspases (z-VAD-FMK). The aforementioned four model proteases, trypsin (serine protease), papain and cathepsin (cysteine proteases), and caspase-8 (caspase), were used to diagnose the specificity and efficacy of the chosen inhibitors when incubating with their preferred substrate. As expected, each specific inhibitor exhibited between 84 and 100% inhibition of protease activity for its given substrate (Fig. S2). Interestingly, papain and cathepsin, a cysteine peptidase of the papain protease family, were both completely inhibited (99–100%) by zVAD-FMK, a pan-caspase inhibitor. This observation was not entirely surprising since it has been suggested that zVAD-FMK can indeed inhibit papain and papain-like peptidases (Rozman-Pungercar *et al*., 2003). However, unlike *H. volcanii* extracts, neither purified papain nor cathepsin demonstrated preferential catalytic cleavage of caspase tetrapeptide recognition sequences (Fig. 1 and Schotte *et al*., 1999).

**Fig. 3:**
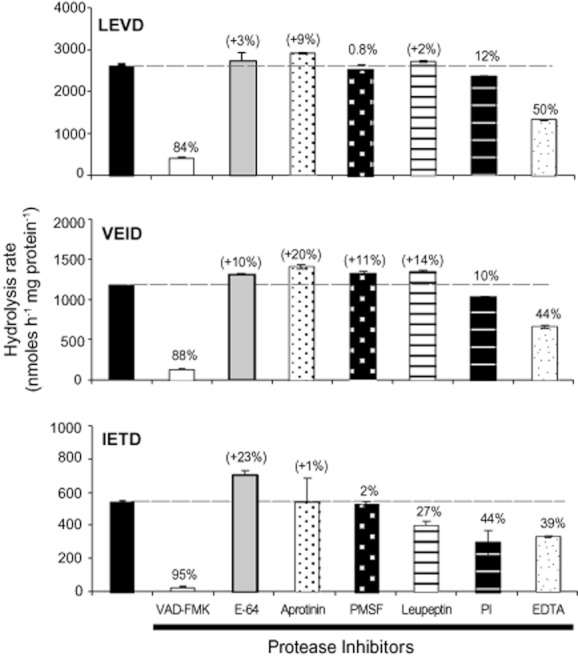
Selective inhibition of caspase activity in *H. volcanii* cell extracts. The efficacy of various protease inhibitors was tested against the cleavage of canonical fluorogenic caspase tetrapeptide substrates (IETD-AMC, LEVD-AMC, and VEID-AMC) as another test of the specificity of activity in *H. volcanii* cell extracts. Cell growth, extract preparation, and kinetic cleavage assays were conducted as described in Fig. 1. The panel of protease inhibitors (10 μM final concentration) were added individually to each reaction included: phenylmethylsulfonylfluoride (PMSF), aprotinin, leupeptin, E-64, protease inhibitor ‘cocktail’ (PI), and EDTA (all from Sigma), as well as the pan-caspase inhibitor zVAD-FMK (20 μM final concentration; Enzo Life Sciences) as previously described (Bidle *et al*., 2010). Fluorogenic substrates were added to cell extracts after a 1 h pre-incubation at 42°C with each protease inhibitor treatment and the degree of inhibition (%) was normalized to the measured activities in uninhibited control reactions. The degree of inhibition (%) was normalized to the measured activities in the uninhibited control reactions (black-filled bars; taken as 100% and corresponding to the dashed line in each panel). Error bars represent standard deviations for triplicate measurements.

**Fig. 4:**
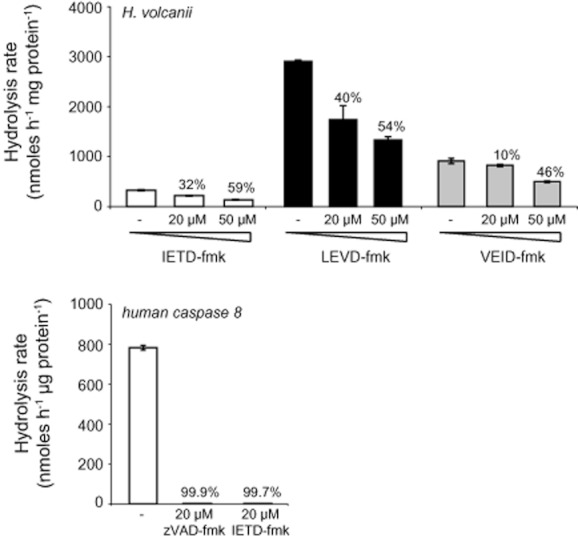
Efficacy of specific fluoromethylketone inhibitors at abolishing caspase-specific activity. Caspase-specific activity in *H. volcanii* cell extracts was measured for IETD-AMC (open bars), LEVD-AMC (black bars), and VEID-AMC (grey bars) in the presence of IETD-fmk, LEVD-fmk and VEID-fmk respectively, at three different concentrations (0, 20, and 50 μM; Enzo Life Sciences). The percent inhibition at each respective concentration (relative to the untreated control) is indicated. For comparison, recombinant human caspase-8 was completely inhibited (> 99%) at 20 μM with both zVAD-fmk and IETD-fmk. Error bars represent standard deviations for triplicate measurements.

We observed nearly complete inhibition (95%) of caspase-8-like activity (IETDase) when *H. volcanii* extracts were treated with zVAD-FMK (Fig. 3), corroborating our previous findings (Bidle *et al*., 2010). Likewise, zVAD-FMK severely inhibited (> 85%) *H. volcanii* caspase-4-like (LEVDase) and caspase-6-like (VEIDase) activities further supporting that this activity is produced by a caspase-like enzyme(s) despite possessing distinct sequence differences to classic caspase superfamily proteins (Bidle *et al*., 2010). Given zVAD-fmk is a broad pan-inhibitor of caspases that is capable of inhibiting different caspases, we further refined our analysis of caspase inhibition by challenging cell extracts with fluoromethylketone inhibitors with higher specificity to individual caspase activities (e.g. LEVD-fmk, VEID-fmk, and IETD-fmk). The response to these specific caspase inhibitors may shed more light on the specificity of the observed caspase activities. In each case, we observed a dose-dependent inhibition of LEVDase, VEIDase and IETDase activities by their respective inhibitors (Fig. 4), but the degree of inhibition with 50 μM of individual inhibitors was considerably lower than that observed for 20 μM zVAD-fmk (Fig. 3). Higher inhibition with zVAD-CHO over specific caspase inhibitors (e.g. IETD-CHO) has also been reported for ‘phytaspases’, plant proteases with caspase activity (Chichkova *et al*., 2010). For comparison, application of zVAD-fmk and IETD-fmk inhibited the activity of purified, recombinant human caspase-8 by 99.9% and 99.7% respectively (Fig. 4). Our findings suggest that the observed caspase activities in *H. volcanii* are not as refined as in higher eukaryotes and may represent a broader, more ancestral type of activity (Fig. 4). These results are also consistent with the presence of either one or several proteins with overlapping activities and/or functions.

**Fig. 5:**
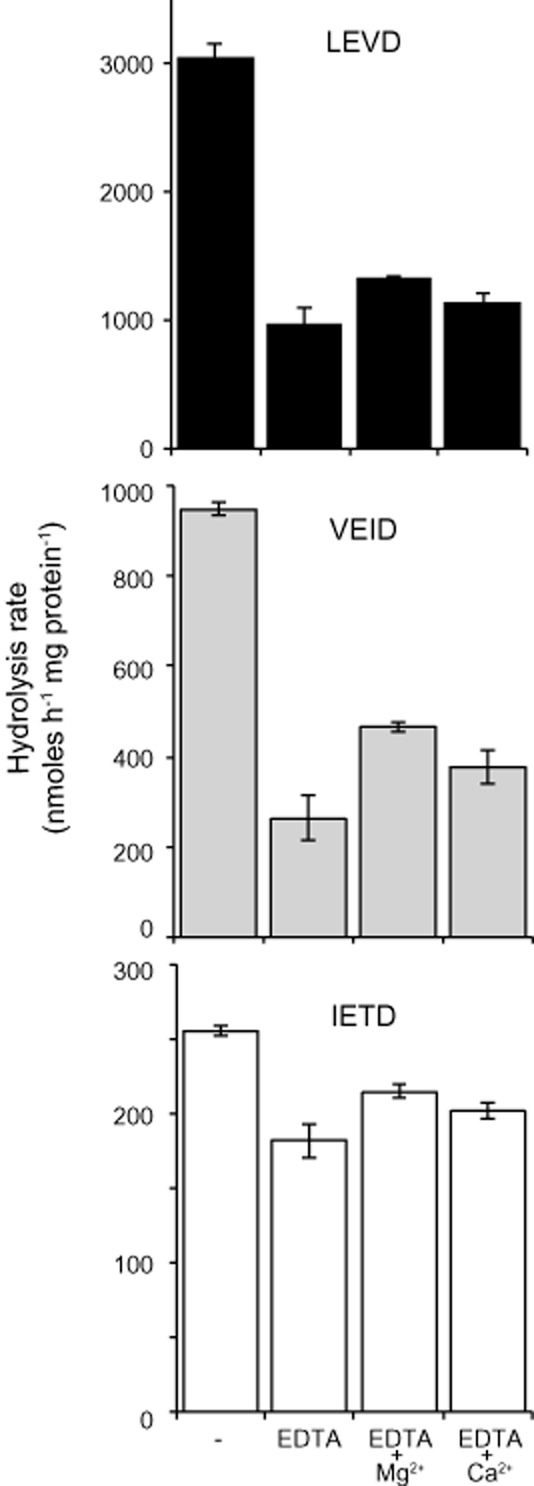
Caspase catalytic activity in *H. volcanii* is stimulated by magnesium. Addition of 100 μM MgCl_2_ to EDTA-treated cell extracts partially reconstituted activity but did not fully recover activity to levels of uninhibited cell extracts (control). Addition of 100 μM CaCl_2_ to EDTA-treated cell extracts did not stimulate caspase activity in EDTA-treated extracts to the same degree. MgCl_2_ and CaCl_2_ were added to EDTA-treated cell extracts 1 h prior to the addition of fluorogenic substrates (LEVD-, VEID-, and IETD-AMC; indicated). Error bars represent standard deviations for triplicate measurements.

Interestingly, EDTA partially inhibited the observed caspase activities derived from all three substrates tested (by 40–50%), indicating a possible requirement for magnesium ions (Rodriguez-Valera, 1995). *Haloferax volcanii* has a very high tolerance for MgCl_2_ (Mullakhanbhai and Larsen, 1975), likely reflective of the abundant MgCl_2_ concentrations found in the Dead Sea, which constitute 50.8% of the anhydrous chlorides on a weight percentage basis, compared with 30.4% for NaCl (Steinhorn, 1983). This is remarkably different from the composition of ocean seawater which is ∼ 97% NaCl. Addition of 100 μM MgCl_2_, to EDTA-treated cell extracts partially reconstituted caspase activity levels for all three substrates tested, but it was not able to restore all activity. MgCl_2_ addition consistently elevated activity more than the addition of 100 μM CaCl_2_ (Fig. 5) albeit slightly, thereby providing some support for a Mg^2+^ requirement.

### Potential cellular roles

Our findings firmly root extremely high levels of specific caspase activity, particularly resembling caspase-4, as the dominant proteolytic activity in this extreme halophilic archeaon. These findings, along with our previous work that linked very high basal caspase activity to the cellular salt stress response (Bidle *et al*., 2010) raise intriguing questions as to the identity, function, and evolution of these caspase-associated proteins. We suggest that this caspase-activity may have emerged in *Archaea*, for essential housekeeping functions and possibly served as a foundation for stress and PCD pathways in higher organisms. In higher eukaryotes, caspase-4 activity has been implicated in endoplasmic reticulum (ER) stress (Kim *et al*., 2006; Binet *et al*., 2010), which can activate the ‘unfolded protein response’ (UPR) under conditions that alter protein folding or calcium homeostasis (Patil and Walter, 2001). UPR leads to the increased expression of chaperones and folding enzymes, which prevent the aggregation of misfolded proteins and facilitate proper protein folding. UPR can also induce components of the protein degradative machinery to remove misfolded proteins, which are tagged by ubiquitin and degraded in the proteosome. This physiological response affords the cell time to survive a stressful insult, unless enhanced or prolonged stress initiates PCD. Interestingly, eukaryotic proteosomes have been shown to contain caspase-like sites and associated activity (Kisselev *et al*., 2003; Murata *et al*., 2009). Archaeal proteosomes, which demonstrate a high level of structural similarity to those found in eukaryotes (Maupin-Furlow *et al*., 2005) have been well studied in *H. volcanii* and play an integral role in the organisms' cellular stress response (Zhou *et al*., 2008). Could the high-level caspase activity be linked to proteosomes and an ancestral UPR response? A UPR is present in all eukaryotes studied to date, but little is known if a similar response operates in *Archaea*. Regulation of proper protein folding in the face of very high extra- and intracellular salt environments encountered during growth is a critical concern for *H. volcanii* (and other extreme halophiles). Our results highlight the need to identify the protein(s) that confer caspase activity and function. Given the high specificity of *H. volcanii* to caspase catalytic activity our findings strongly suggest that recently developed in situ inhibitor trapping techniques (Tu *et al*., 2006; Mohr and Zwacka, 2007) are a promising avenue to identify these enigmatic proteins and begin to elucidate their physiological roles and molecular evolution.

## Supporting information

**Fig. S1.** Panel of proteolytic activities in partially purified protein fractions. Hydrolysis rates were determined for cell extracts (CE) from exponentially growing *H. volcanii* cells, partially purified proteins obtained by sequential ammonium sulfate (AmSO_4_) precipitation (20%, 40%, 60%, 80% and 100%), and non-AmSO_4_-precipitable proteins (soluble). AmSO_4_ was sequentially added to cell extracts at 4°C with constant rocking and incubated for 30 min at each step. Prior to each AmSO_4_ additon, proteins were pelleted at 10 000 g. The supernatant, representing dissolved proteins, was transferred to a new tube and used for the next percent AmSO_4_ addition. Protein pellets were resuspended in 100 mM Tris-Cl (pH 7.4)/150 nM NaCl and activity was measured using the aforementioned assay conditions and panel of fluorogenic substrates. Note difference in scale bewteen caspase substrates (upper panel) and other protease substrates (lower panel). Hydroysis of Leu-AMC was also measured but no activity was detected in any fraction. Error bars represent standard deviations for triplicate measurements.

**Fig. S2.** Protease inhibitor efficacy on model protease substrates. To verify inhibitor effectiveness, purified proteases (recombinant human caspase-8, trypsin, papain, cathepsin B) were individually incubated with inhibitor treatments and compared with uninhibited control reactions. Each purified enzyme was pre-incubated with individual protease inhibitors at 37°C for 1 h at the aforementioned concentrations (see Fig. 3) prior to the addition of the preferred fluorogenic substrate for each respective enzyme: caspase-8 with IETD-, trypsin and cathepsin with ARR-, and papain with LLVY-AMC.
